# Giant Pheochromocytoma Diagnosis Confounded by Amphetamine Use

**DOI:** 10.1155/2023/8799089

**Published:** 2023-01-27

**Authors:** Shreya Amin, Matthew Gilbert, Kaitlyn Barrett

**Affiliations:** Department of Medicine, University of Vermont, Division of Endocrinology, Burlington, Vermont, USA

## Abstract

**Objective:**

Diagnosis of giant pheochromocytoma is difficult; patients often lack the classic triad and presence of gross biochemical positivity. At times, presence of sympathetic stimulant drugs can further complicate the clinical picture. Here, we present a case of giant “functional” pheochromocytoma with a history of amphetamine use. *Case Description*. 37-year-old female presented with a 1-day history of abdominal pain. CT abdomen identified a 12.5 cm heterogeneously enhancing left adrenal mass. Plasma/urine catecholamine and metanephrine levels were markedly elevated with evidence of elevated serum/urine cortisol. However, the patient's subsequent urine toxicology was found to be positive for amphetamines, which she later admitted to using, 1 week prior to admission. Repeat biochemical workup after 1 week drug washout period showed improvement in both catecholamine and cortisol levels. Given the high degree of suspicion for PCC, an open laparoscopic adrenalectomy was performed with histology confirming SDHB gene mutation positive giant pheochromocytoma. *Discussion*. Diagnosis of PCC in a patient with a history of amphetamine abuse remains an enigma, to which addition of it being a giant PCC that are rare and typically silent further confounds the clinical picture as seen in this case.

**Conclusion:**

PCC could be termed a “chameleon” tumor given its varied clinical presentations and lack of standardized biochemical and radiological data (giant, pheochromocytoma, and amphetamine).

## 1. Introduction

Pheochromocytomas (PCCs) are rare catecholamine producing tumors with lethal effects if not diagnosed and treated in a timely manner. On the other hand, surgically treated PCCs have an almost 90% 5-year and 78.9% 10-year survivability rate, as reported in a large Denmark-based study [1].

The biggest limitation in PCC management remains its diagnoses as classic triad are often paroxysmal [2] and hypertension, seen in 90% of PCC cases, could either be sustained, paroxysmal, or in some cases normotensive or hypotensive [3]. Several other endocrine conditions such as hyperthyroidism, neuroendocrine tumors, and carcinoid syndrome can present as PCC symptomatology mimickers [4]. Other nonendocrine-related symptom mimickers include anxiety, panic disorder, sympathomimetic drugs ranging from OTC pseudoephedrine, prescription or illicit amphetamines, SNRI, SSRI, or illicit cocaine [4, 5]. These drugs not only mimic PCC symptomatology but also are a biochemical confounder [5, 6].

Giant pheochromocytomas are categorized as tumors >7 cm in size [3, 7]. These giant pheochromocytomas are reported to be rare with just a few case reports published in literature over the past two decades. Despite their size, these giant PCCs are often clinically silent. They appear cystic in imaging and often present clinically with abdominal pain or within incidental imaging findings [3, 7, 8].

## 2. Case Report

**Figure 1:**
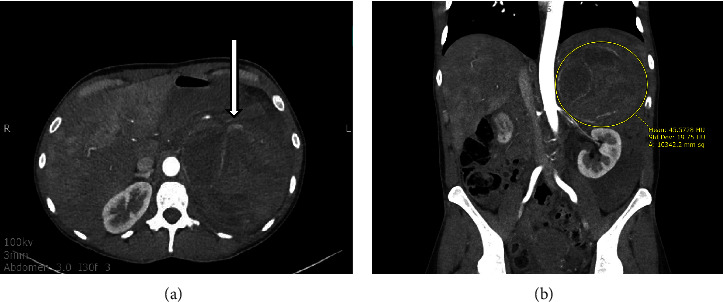
CT abdomen/pelvis with contrast showing a heterogenous 12.5 cm × 10.7 × 9.4 cm left adrenal mass coronal view (a) and axial view (b).

A 37-year-old female with a past medical history of polysubstance abuse and generalized anxiety disorder (GAD) presented to ED for sudden onset of abdomen pain for 1 day. CT abdomen/pelvis with contrast showed a 12.5 cm heterogenous enhancing left adrenal mass with mild to moderate hyperdense fluid throughout the abdomen from a presumed previous hemorrhage (Figure 1). The patient was hemodynamically stable with a hemoglobin of 13 g/dL. CT angiography ruled out active extravasation.

The patient denied any history of hypertension, diaphoresis, but did report occasional headaches, palpitations, and a 10–15 lbs weight loss over the last 4 months. She denied any current substance use. Medication history included suboxone and Wellbutrin. Family history was otherwise negative for any endocrine disorders.

Hemodynamically she had mild tachycardia with HR 112 bpm, BP 114/92 mmHg, temp 98.6°C, respiratory rate of 18 breaths per min, and an O_2_ sat of 99% on room air. On physical examination, she was found to have abdominal distention and diffuse abdominal pain with diffuse anasarca. A focused exam was negative for any cushingoid features. The biochemical workup revealed plasma cortisol 6 times ULN, plasma metanepherines 25–30 times ULN, and urine metanepherines 30–100 times ULN. Detailed lab values and normal reference ranges for our institution are reported in Table 1. Urine toxicology obtained was positive for amphetamines. With this finding, the patient now admitted to amphetamine abuse 1 week prior to her admission. Given the biochemical evidence of hypercortisolism and adrenergic surge, which can be seen with amphetamine use and to lesser degree with SNRI use [5, 9, 10], repeat biochemical workup was ordered in 1 week to allow for a drug washout. 2 weeks since her last reported amphetamine use, serum cortisol declined by >50%, and similarly the urine metanephrines levels, although still elevated, were now only 20–40 times ULN compared to previous 30–100 times ULN (Table 1).

Given the still posed diagnostic dilemma, an MIBG scan was ordered, however due to institutional limitations, could not be conducted. Differential diagnosis at this point, in order of most to least likely, included giant PCC, large corticomedullary tumor, and nonfunctioning adrenal adenoma with substance induced hypercortisolemia/catecholamine surge.

The decision regarding optimal preoperative management was complicated by an unclear tumor type. However, given higher probability of PCC, she was started on 1 mg doxazosin twice daily with a maximum tolerated dose of 6 mg twice daily, 12.5 mg metoprolol twice daily with a max dose of 25 mg twice daily. This did cause the patient's BP to drop, systolic BP in 80s, which was treated with aggressive 1-2 L intravenous fluid hydration (IVF) and 1-2 g sodium tablets per day. She then started to third space likely due to poor albumin reserve related to malnourishment (albumin 2.5 on presentation), requiring albumin infusion.

An open laparotomy was performed which showed a rock-hard mass with associated inflammation and hemorrhaging involving the adrenals, spleen, and tail of pancreas. A left adrenalectomy, splenectomy, and distal pancreatectomy was performed. The patient's intraoperative and postoperative vitals remained stable and she was safely titrated off both beta and alpha blockers day two postoperation. The surgical pathology identified a 10.5 cm left giant pheochromocytoma with a mixed hemorrhagic and cystic center. It was well encapsulated within the fibrous. No lymphovascular or fat invasion was observed. Immunohistochemical staining was positive for chromogranin A and INSM1 with loss of SDHB expression in tumor cells. No metastasis was identified in pancreatic or splenic tissue. Patient's plasma metanephrines improved to normal by day 3 (Table 1).

She is now being monitored periodically for recurrence of disease with surveillance biochemical workup and CT abdomen/pelvis due to positive SDHB mutation.

## 3. Discussion

Catecholamines such as epinephrine, dopamine, and norepinephrine are released by chromaffin cells within the adrenal medulla. An excess presence of these cause symptoms of headaches, palpitations, and diaphoresis [2, 4]. This classic PCC triad is only seen in 30% of cases [11] with atypical symptoms seen in 9-10% of cases [12]. Few case reports have identified myocardial infarction, stoke, or DKA [11, 12] as initial presentations for PCC. Thus, clinical suspicion for PCC based on symptoms alone can be challenging and therefore, presence of gross biochemical positivity is often expected to provide clarity.

Medications such as SSRI/SNRI's and amphetamines can increase extracellular catecholamine levels [5, 9]. Amphetamines directly redistributes catecholamines from synaptic vesicle to cytosol [9] as well as can cause symptoms of hypertension, diaphoresis, headache, and palpitations related to catecholamine excess [9]. Therefore, amphetamines use can mimic PCC symptomatically and also cause gross biochemical catecholamine excess making differentiating it from PCC perplexing, as seen in our case.

Given posed diagnostic dilemma, imaging modalities are often used next to improve the diagnostic sensitivity. However, even on imaging, PCCs may have atypical presentations as fatty, cystic, or calcified masses with variable signaling on T2 MRI and variable degree of contrast washout on CT [13]. Functional imaging such as MIBG is 95–100% specific for PCC; however, it typically has lower sensitivity at 75–90% [13]. Secondary centers such as ours may often run into logistics issues limiting availability of MIBG scan.

One could assume that larger PCCs would be associated with higher catecholamine release and thus present with florid symptoms associated with catecholamine surge making diagnosis of PCC easier. On the contrary, to the best of our knowledge all giant pheochromocytomas reported in the literature thus far have been silent with mild catecholamine elevation often presenting as incidental findings on imaging or with abdominal pain due to mass effect [14–16]. The reasons for this have been postulated as encapsulated catecholamines within the tumor wall preventing their systemic release or inner tissue necrosis due to PCCs being hyper vascular limiting catecholamine synthesis [8]. Despite being encapsulated and with inner necrosis, gross catecholamine excess was seen in our patient from her giant PCC.

We presented here a unique case of a giant 10.5 cm “functional” pheochromocytoma with several diagnostic dilemmas posed by atypical presentation and amphetamine abuse history. Although initially seen grossly positive urine/plasma catecholamines and metanephrines levels made diagnosis of PCC a high probability, presence of concomitant hypercortisolemia and >50% decline in catecholamines and cortisol levels after 1 week of drug washout period added further complexities around diagnosis. The diagnosis of PCC was confirmed after the tumor resection and improvements in her catecholamine levels after tumor resection (Table 1).

## 4. Conclusion

Giant PCCs are rare entity in itself but those reported are typically silent, thereby making our case the first one to report a functional giant PCC. Diagnosis of “functional” giant PCC would have otherwise been straight forward had it not been for our patient's use of amphetamines.

## Figures and Tables

**Table 1:** Summary of patients' laboratory evaluation data during initial workup, after 1 week of drug washout period, and following surgical removal of her tumor.

	Initial	Drug washout	Postoperation day 3 (follow-up)	Normal reference range
Plasma metanephrine (nmol/L)	15	8	<0.2	<0.50 nmol/L
Plasma normetanephrine (nmol/L)	26	13	0.7	<0.9 nmol/L
Urine metanephrine (mcg/24 hr)	18850	8477	390	149–535 mcg/24 hr
Urine normetanephrine (mcg/24 hr)	11760	7584	1992	111–419 mcg/24 hr
Urine total metanephrine (mcg/24 hr)	30310	16051	2382	3–180 mcg/24 hr
24 hr urine cortisol (mcg/24 hr)	180			3.5–45 mcg/24 hr
Random serum cortisol (*μ*g/dL)	104	35	22	2–14 *μ*g/dL

## Supplementary Materials

Supplementary Materials List of abbreviations.
