# Supplementary material for: Giant Pheochromocytoma Diagnosis Confounded by Amphetamine Use
Source: Case Rep Endocrinol. 2023 Jan 27;2023:8799089. doi: 10.1155/2023/8799089 (PMC9897925; doi:10.1155/2023/8799089)
Supplement: Supplementary Materials — List of abbreviations. [file 8799089.f1.docx]

List of abbreviations

1. BP:- Blood pressure
2. ED:- Emergency department
3. GAD:- Generalized anxiety disorder
4. PCC:- Pheochromocytoma
5. SNRI:- Serotonin Norephinephrine reuptake inhibitor
6. SSRI:- Selective Seratonin reuptake inhibitor
7. OTC:- Over the counter
